# DISE/6mer seed toxicity-a powerful anti-cancer mechanism with implications for other diseases

**DOI:** 10.1186/s13046-021-02177-1

**Published:** 2021-12-10

**Authors:** Ashley Haluck-Kangas, Monal Patel, Bidur Paudel, Aparajitha Vaidyanathan, Andrea E. Murmann, Marcus E. Peter

**Affiliations:** Division Hematology/Oncology and Department of Biochemistry and Molecular Genetics, Feinberg School of Medicine, Northwestern University, 303 East Superior Street, Lurie 6-123, Chicago, IL 60611 USA

**Keywords:** microRNAs, RNA interference, Cell death, DISE

## Abstract

micro(mi)RNAs are short noncoding RNAs that through their seed sequence (pos. 2–7/8 of the guide strand) regulate cell function by targeting complementary sequences (seed matches) located mostly in the 3′ untranslated region (3′ UTR) of mRNAs. Any short RNA that enters the RNA induced silencing complex (RISC) can kill cells through miRNA-like RNA interference when its 6mer seed sequence (pos. 2–7 of the guide strand) has a G-rich nucleotide composition. G-rich seeds mediate 6mer Seed Toxicity by targeting C-rich seed matches in the 3′ UTR of genes critical for cell survival. The resulting Death Induced by Survival gene Elimination (DISE) predominantly affects cancer cells but may contribute to cell death in other disease contexts. This review summarizes recent findings on the role of DISE/6mer Seed Tox in cancer; its therapeutic potential; its contribution to therapy resistance; its selectivity, and why normal cells are protected. In addition, we explore the connection between 6mer Seed Toxicity and aging in relation to cancer and certain neurodegenerative diseases.

## Background

In this review, we will summarize what is currently understood about a novel mechanism that regulates cell fate, 6mer Seed Toxicity. We posit that 6mer Seed Tox evolved to be a powerful anti-cancer mechanism with potential relevance to the etiology of other diseases. We usually refer to cancer as the disease that patients present with in the clinic. However, cancer begins at the single cell level. A single cell may accumulate mutations and become neoplastically transformed. The acquisition of mutations and subsequent genomic instability is one of the central hallmarks of cancer [1]. Cancer only manifests in multicellular organisms likely because cell division is a big risk factor for the accumulation of mutations [2]. We propose that throughout the ~ 2 billion years of evolution of multicellular organisms very powerful mechanisms arose to eliminate cancerous cells. To be effective, this elimination would need to be impervious to the development of resistance. The immune system can be argued to serve this function; particularly in light of the recent successes using immune checkpoint therapy to treat cancer [3]. However, the immune system developed relatively recently - only about 500 million years ago [4] and immune deficient mice and people do not usually succumb to massive tumor formation in multiple organs. While immune cells can be turned against cancer cells, their anti-cancer function does not appear to be a major mechanism through which cancer formation was prevented throughout the evolution of multicellular organisms. This is consistent with the observation that cancer cells can become resistant to the antitumor activity of the innate immune system (e.g., the activity of interferons, [5]) or the adaptive immune system (e.g., induction of apoptosis in tumor cells, [6]). However, there is a much more archaic mechanism that may also have evolved as an early immune-like system, RNA interference (RNAi), an RNA guided mechanism for sequence-specific gene silencing. RNAi likely evolved to fight endogenous and exogenous RNA-based viruses, but has developed multiple functions and become essential to cells [7]. In this review, we will discuss a novel RNAi-based anticancer mechanism that we argue evolved to effectively eliminate cancerous cells. This kill mechanism functions through the targeting of a redundant network of genes by a small number of often non-redundant components which are essential to the survival of all cells. Despite the enormous capacity of cells in a heterogeneous tumor to mutate and change, a tumor is unlikely to find a way around this novel mechanism of death. Based mostly on our own work, we will describe the discovery of this system, propose a model for its cancer selectivity, outline a path to develop this concept into a general anti-cancer treatment, and finally discuss its relevance to other diseases in the context of aging.

### microRNAs, their biogenesis, and the evolution of RNA interference

RNAi is mediated by micro (mi)RNAs, small (18-22 nt long) noncoding RNAs that are generated from long double-stranded (ds)RNAs (15). miRNAs are transcribed in the nucleus as hairpins that are processed by the Drosha/DGCR8 microprocessor [8, 9], and transported into the cytosol by Exportin-5 [10]. In the cytosol, dsRNA is cleaved from the hairpin by the Dicer/TRBP complex [11, 12]. This mature miRNA is then loaded into the RNA induced silencing complex (RISC) by binding to Argonaute (Ago) proteins [13]. One of the strands of the miRNA (the guide strand) stays in the RISC, while the other (the passenger strand) is degraded [14]. In the RISC, the guide strand may target hundreds of mRNAs through binding of the seed region (position 2–7/8) to complementary seed matches, mostly located in the 3′ untranslated region (3′ UTR) of the targets [15, 16]. This results in translational repression through multiple mechanisms [17].

Both miRNAs and RNAi pathway genes are highly conserved throughout evolution. The human genome codes for more than 2300 miRNAs [18] and some human miRNAs are estimated to be ~ 800 million years old [19]. Components of the RNAi pathway may be even older and are conserved across metazoans [20]. Ago proteins are even found in certain unicellular budding yeast species [21]. The conservation of miRNAs and components of the miRNA pathway reflect the essentiality of RNAi for cell survival. In fact, loss of genes required for miRNA biogenesis and RISC formation are mostly deleterious to cells. This is true for Drosha, Dicer [22], and the Ago proteins. In fact, when all four Ago genes are deleted no cell type, including embryonic stem cells, can survive [23]. Due to the essential nature of RNAi, it is unlikely that cancer cells can bypass this mechanism. Thus, an anti-cancer mechanism mediated by RNAi is attractive for the treatment of therapy resistant cancers.

### The discovery of DISE/6mer seed toxicity

In 2014, we reported that a number of siRNAs and shRNAs designed to silence either the death receptor CD95/Fas or its ligand, CD95L, killed all tested cancer cells by activating multiple cell death pathways. No pharmacological inhibitor or knockdown of any single gene was found to block this form of cell death. Repeat treatment with sh/siRNAs was effective in killing, suggesting that cancer cells could not easily develop resistance [24]. Subsequently, we demonstrated that these and many other si/shRNAs killed cells even in the absence of the mRNA they were designed to target [25, 26]. A detailed analysis of the requirements for this dsRNA toxicity revealed that they indeed killed cells through canonical RNAi, requiring a functional RISC. In contrast to the function of siRNAs, they only relied on position 2–7 of the guide strand, which is the minimum seed sequence required for a short RNA to act as a miRNA [25]. It became clear that the phenomenon we observed was intrinsically linked to the function of miRNAs. The study also revealed that the tested si/shRNAs killed cells by causing downregulation of a number of genes that were found to be critical for cell survival in a number of gene essentiality screens [25]. Not only were the targeted mRNAs enriched in cognate seed matches for the expressed toxic siRNAs, but targeting these mRNAs individually using siRNA SmartPools killed cells [25]. More recently we confirmed that mutating seed matches in selected targeted survival genes prevented silencing of these genes by such a toxic siRNA, but not toxicity [27]. Based on the mechanism of action, we called this mechanism of cell death, Death Induced by Survival gene Elimination (DISE) [28].

Using chimeras of toxic and nontoxic siRNAs we then determined that indeed only a 6mer seed was necessary to induce DISE [25]. We therefore conducted an arrayed screen designed to identify the composition of the most toxic of all possible 4096 6mer seeds. The seeds were embedded in a neutral siRNA backbone. To ensure that the desired seed was tested, the designated passenger strand was blocked from entering the RISC by adding 2′-O-methylation groups to nucleotide positions one and two [29, 30]. We screened all 4096 siRNAs in three human and three murine cell lines ([27, 30], 6merdb.org). The screen revealed that the rules of toxicity were very similar in all cell lines suggesting that this kind of toxicity kills cancer cells regardless of the tissue or species of origin [27, 30]. Across all cell lines the most toxic of all 6mer seeds had a high G-content with the most toxic seeds carrying Gs at the 5′ end and a C at the 3′ end of the 6mer seed (position 7 of the siRNA). Further validating the concept of DISE, we determined that these toxic seed containing siRNAs targeted a large set of critical survival genes in a miRNA-like fashion. The toxic G-rich seeds targeted C-rich seed matches, which were enriched at the beginning of the 3′ UTRs of target mRNAs [30].

The DISE/6mer Seed Tox concept was recently confirmed in the context of prostate cancer [31]. Similar to our observations with CD95/CD95L, Corbin et al. found that a number of sh- and siRNAs designed to target the androgen regulated tumor suppressor, TMEFF2, killed prostate cancer cells by targeting genes distinct from the gene they were actually designed to target. The authors then discovered that many of these si-/shRNAs targeted a network of survival genes in their 3′ UTR through 6mer Seed Tox. This Androgen Network Death Induced by Survival gene Elimination (AN-DISE) could also be induced by two of the CD95 and CD95L targeting shRNAs we had described, shR6 and shL3. These data suggest that in androgen receptor (AR) signaling dependent prostate cancer cells the network of targeted survival genes is dominated by AR regulated genes.

Another recent report validating the 6mer Seed Tox concept came through the study of dual function mi/siRNAs, which were generated based on AGO-CLIP data [32]. These siRNAs were designed to target oncogenes through complete complementarity and at the same time these siRNAs were selected to contain 6mer seed sequences of major tumor suppressive miRNAs. Similar to siRNAs that act through 6mer Seed Tox, these designed siRNAs were shown to target hundreds of seed matches in tumor promoting genes. The authors discovered that three of the four tested siRNAs were toxic to cancer cells through 6mer Seed Tox by targeting a network of survival genes.

### Most abundant miRNAs do not target genes that are critical for cell survival

**Fig. 1:**
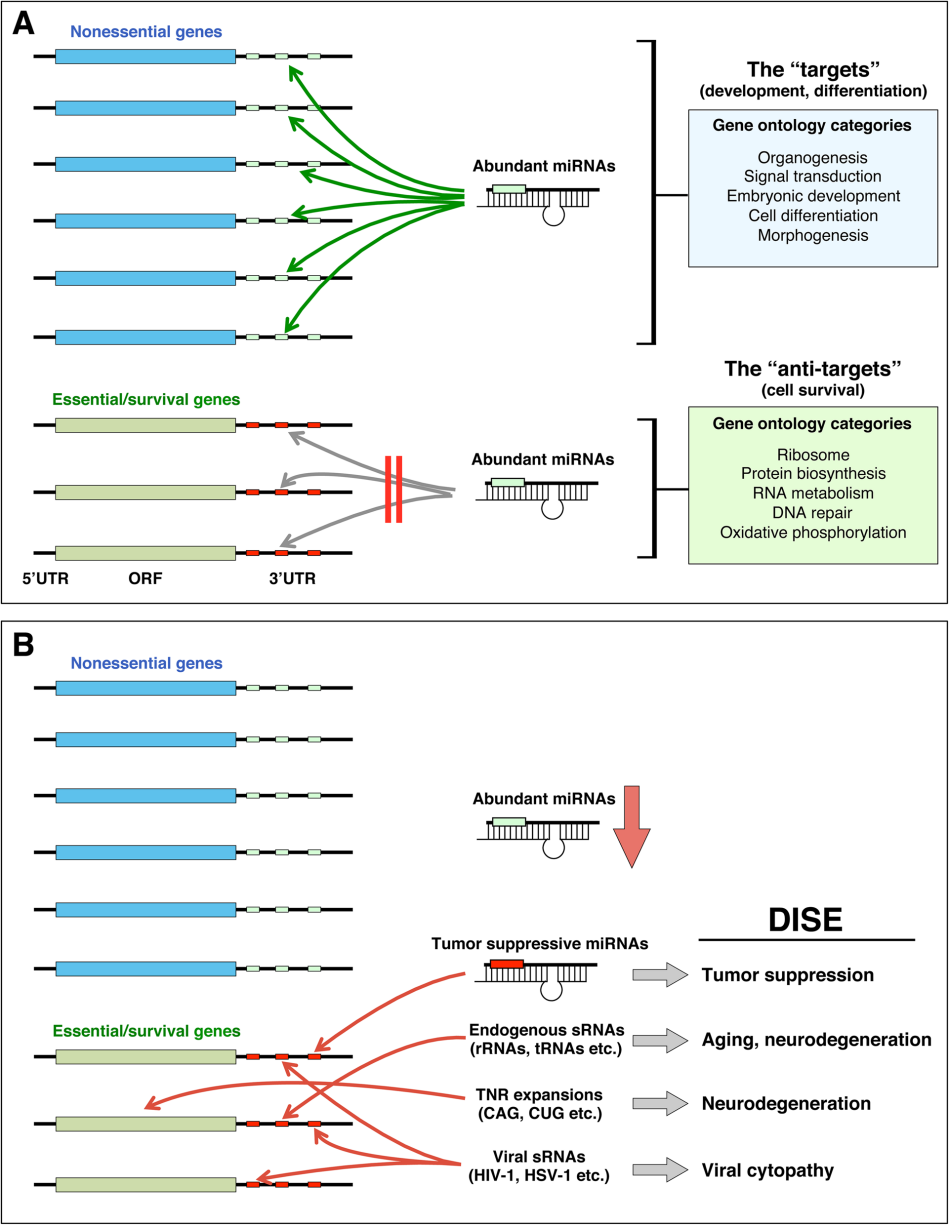
Model to illustrate the role of DISE/6mer seed toxicity in cancer and other diseases. **A** High expression of miRNAs with nontoxic seeds. *Left:* The genome contains ~ 20,000 genes (blue and green genes) of which about ~ 10% are essential for the survival of all cells (green genes), as determined by various lethality screens. Almost all genes contain 3′ UTRs, the predominant location of seed matches targeted by miRNAs. The most abundant miRNAs target seed matches in the 3′ UTR of nonessential genes (light green boxes). Consistently, genes containing seed matches for these highly abundant miRNAs (the ‘targets”), function in biological processes such as development and differentiation (*top right*) [36]. If such highly abundant miRNAs were to target seed matches in the 3′ UTR of essential survival genes (red boxes), that would result in the death of the cell (*bottom left*). Consistent with this model, genes devoid of seed matches of highly abundant miRNAs (the “anti-targets”) fall into gene ontology terms that are consistent with cell survival, including protein biosynthesis, DNA repair, and DNA metabolism (*bottom right*) [36]. **B** Low expression of miRNAs with nontoxic seeds. Under conditions in which the most abundant miRNAs with nontoxic seeds are lost, specialized miRNAs with G-rich toxic seeds (e.g., miR-34a-5p), which are often tumor suppressive, target C-rich seed matches (red boxes) in the 3′ UTR of essential survival genes (green genes), inducing DISE. When the expression of protective nontoxic miRNAs is reduced sRNAs other than miRNAs can enter the RISC and depending on the type of sRNA and the tissue, this loading of sRNAs could result in degeneration through DISE induction (*bottom right*). These could be rRNA or tRNA fragments which have been shown to be upregulated in AD [37–39] and aging [40], TNR based sRNAs as shown in multiple diseases including HD, or even sRNAs from pathogens such as viruses. UTR, untranslated region; ORF, open reading frame; DISE, death induced by survival gene elimination; rRNA, ribosomal RNA; tRNA, transfer RNA; TNR, trinucleotide repeat

The human genome codes for ~ 20,000 genes. According to a number of genome-wide lethality and fitness screens at least 1000 of them are critical for the survival of cells [33–35]. We refer to these as essential/survival genes (Fig. 1A, bottom). The majority of genes, however, are not essential genes but regulate everything from development to metabolism, to general cell signaling (Fig. 1A, top). While there is evidence that miRNAs target mRNAs in the coding region [41, 42], most miRNAs function by targeting seed matches located in the 3′ UTR of genes. Consistent with this conclusion all miRNAs and siRNAs carrying toxic 6mer seeds we tested targeted seed matches located predominantly in the 3′ UTR [25, 27, 30]. Most genes contain 3′ UTRs, including survival genes. In fact, the average length of the 3′ UTR of a list of curated survival genes compared to all nonsurvival genes revealed that the 3′ UTR of survival genes is significantly longer than that of nonsurvival genes (unpublished data). This would suggest that survival genes can also be miRNA targets.

Major miRNA families are upregulated late during embryonic development, often to maintain lineage commitment and tissue differentiation, and remain highly expressed in most tissues [43]. Based on these observations we postulate that the 3′ UTR of survival genes should not contain seed matches that are targeted by highly expressed miRNAs, which otherwise would kill cells through 6mer Seed Tox (Fig. 1A, bottom). The avoidance of miRNA seed matches by certain classes of genes was previously postulated in a report studying the coevolution of miRNAs and 3′ UTRs [36]. In this study the authors classified genes that contained miRNA seed matches as “targets” and the ones that did not as “anti-targets”. Interestingly, a gene ontology analysis of these two groups of genes revealed the genes in the target group to be involved in many developmental, differentiation and general signaling related processes. In contrast, the anti-target genes were found to be involved in processes consistent with cell survival (Fig. 1A, right). Our recent Seed Tox analysis of small RNA Seq data showed that several normal tissues have an abundance of miRNAs with nontoxic seeds [44]. Based on our findings we now conclude that most highly expressed miRNAs carry nontoxic 6mer seeds that are devoid of Gs.

### A class of conserved tumor suppressive miRNAs kill cancer cells through DISE/6mer seed toxicity

Based on our data, we predicted the existence of specialized endogenous miRNAs that carry G-rich seeds. We hypothesized that these may have evolved to be tumor suppressive. Indeed, many well characterized tumor suppressive miRNAs contain Gs in their seed [44].

miR-[34a/34b/34c/429b]-5p (the miR-34-5p family) is a master tumor suppressive miRNA family that was found to exert its tumor suppressive function, at least in part, through 6mer Seed Tox [30]. Its 6mer seed, GGCAGU starts with two Gs, which our screening data suggests to be highly toxic to cells (see 6merdb.org). To determine how much of miR-34a-5p’s cancer killing activity came from just its 6mer seed, we expressed the mir-34a-5p seed sequence in a neutral siRNA scaffold and compared its activity to that of the full length miR-34a-5p. Both activities were highly similar with ~ 80% of the same genes (many of them survival genes) downregulated cancer cells transfected with either RNA duplex [30]. These data suggest that the miR-34-5p family employs 6mer Seed Tox to kill cells. This conclusion is supported by the predicted function of the genes (~ 700) that have been identified as miR-34a-5p targets. They are predominantly involved in regulating cell survival and drug resistance (discussed in [30]). miR-34a-5p therefore fulfilled the criteria of being a DISE-inducing miRNA that arose during evolution to kill cancer cells. More recently we found that another major tumor suppressive miRNA family, miR-[15a/15b/16]-5p (the miR-15/16-5p family), also kills cancer cells in part through 6mer Seed Tox [45]. While not as potent as the miR-34-5p family, we estimated through 65% overlap of down-regulated target genes, again including many survival genes, that most of the cytotoxic activity of miR-15/16-5p stems from targeting mediated by its 6mer seed.

However, not all tumor suppressive miRNAs engage in 6mer Seed Tox. Major miRNA families such as let-7 and miR-200 do not carry toxic 6mer seeds, and instead exert their tumor suppressive activity by inducing and/or maintaining cell differentiation [46–49], or in the case of miR-200 epithelial differentiation, specifically [50–52].

The identification of endogenous tumor suppressive miRNAs that are toxic to cells through 6mer Seed Tox suggests that certain miRNAs are purposefully expressed to target essential/survival genes (Fig. 1B). Their toxic activity may be enhanced when expressed in transformed cells due to the global downregulation of normally abundant miRNAs in cancer [53, 54].

### 6mer seed Tox and the evolution of miRNAs

The existence of specialized miRNAs that contain G-rich 6mer seeds raised the question of how toxic miRNAs were selected during evolution, and how to prevent accidental cell death. By analyzing all major ~ 2600 mature human miRNAs we found two important features of miRNAs that fundamentally support the concept that DISE is an evolutionarily conserved process [30]:

1. We observed in sequence logos of miRNAs grouped by age that most younger miRNAs (< 10 million years old) contain Gs as the most abundant nucleotide in five out of the six positions of their 6mer seed [30]. These include a number of miRtrons—miRNAs that are generated through splicing without the involvement of Drosha [55]. In contrast, most older miRNAs contain fewer Gs in their 6mer seeds. Remarkably, the oldest miRNAs (> 800 million years old) contained mostly As and Us as the most abundant nucleotides in all six seed positions.
2. By ranking all mature miRNAs according to their predicted 6mer Seed Toxicity we could efficiently separate oncogenic miRNAs from tumor suppressive ones [30].

Despite the observed selection for more non-toxic miRNAs over time, toxic miRNAs with G-rich seeds have been fixed in the genome. Our analysis revealed that some of the best characterized tumor suppressive miRNAs carry a toxic seed [30]. They are often induced by powerful tumor suppressor proteins (p53 in case of miR-34 [56] and E2F in case of miR-15/16 [57]). Hence, tumor suppressive miRNA expression may be induced during oncogenic cell stress responses, resulting in the loading of these miRNAs carrying toxic seeds into the RISC and cancer cell death.

### Non-canonical short RNAs in the RISC are a source of DISE-inducing short RNAs in cells

Our data suggest that the 6mer Seed Tox concept is not limited to miRNAs. Any short (s)RNA that can enter the RISC and contains a G-rich seed could have anti-cancer activity. We demonstrated this in HCT116 Drosha knock-out (KO) cells which are devoid of most endogenous miRNAs. When we pulled down Ago proteins 1–4 in wild type (wt) and Drosha KO HCT116 cells and sequenced the bound RNAs, as expected, we observed that the RISC content of wild-type HCT116 cells consisted primarily of miRNAs (> 98% of all Ago-bound sRNAs) [37]. In contrast, in cells lacking Drosha we noticed two things: 1) the amount of RISC-bound sRNA was reduced by > 10 fold and 2) of the bound sRNAs only 34% were miRNAs. More importantly, however, the average 6mer seed viability of the RISC-bound reads—predicted based on our 4096 seed screen data—differed. In Drosha KO cells, the 6mer seed viability was much lower (20–40% viability) than in the wt cells (~ 80% viability) [37]. This suggested that endogenous toxic sRNAs entered the RISC and could negatively affect cell growth. Consistently, we reported that Drosha KO cells grew more slowly than parental cells. Knockdown of Ago2 completely corrected this growth defect [58] suggesting that endogenous RISC-bound sRNAs can affect cell fate presumably through 6mer Seed Tox.

A recent study provided evidence that a small noncoding RNA can exert tumor suppressive activities through RNAi. vtRNA2–1/nc886 is a non-coding vault RNA (vtRNA) that is cleaved by Dicer to give rise to a dsRNA. Its 3p arm is predominantly expressed and carries the seed GCGGGU, a 6mer seed sequence that was toxic to all six human and murine cancer cell lines tested in our 6mer seed screen (6merdb.org). Interestingly, vtRNA2–1 is one of the predominant RNA species in the RISC of Drosha KO cells [37]. However, vtRNA2–1 was found to exert a tumor suppressive phenotype even in cells with an intact miRNA biogenesis pathway and expression of endogenous miRNAs [59]. In fact, nc886 was recently shown to act as a tumor suppressor in prostate cancer, however, its expression often is epigenetically suppressed [60].

Many of the sRNAs we detected in the RISC of Drosha KO cells are the fragments of highly abundant cellular RNAs including tRNAs and rRNAs [37]. These RNAs play important structural roles, carry extended G/C-rich stem regions, and fragments of these RNAs which form naturally (often in response to stress [61, 62]) have been shown to enter the RISC [62, 63]. According to our screen, any such RNA fragment that carries G-rich nucleotides in position 2–7 is expected to exert toxicity through the induction of DISE. Thus, we expect that many fragments of tRNAs, rRNAs, and vtRNAs will exert toxicity through DISE when loaded into the RISC. Conversely, we postulate that highly abundant miRNAs with nontoxic seeds play at least three different roles: 1) They regulate biological processes such as differentiation and development, 2) by occupying the RISC they prevent miRNAs with toxic seeds from entering, and 3) they protect cells from small fragments of highly abundant and often G or C-rich endogenous RNAs, inducing tRNAs, rRNA, vtRNAs, snoRNAs and others.

### Use of 6mer seed Tox in cancer therapy

We have provided the first evidence of the power of harnessing 6mer Seed Tox for cancer therapy. In an orthotopic mouse xenograft model of ovarian cancer we injected intraperitoneally (i.p.) two CD95L-derived DISE-inducing siRNAs coupled to HDL mimetic nanoparticles [64]. Repeated treatment resulted in reduced tumor growth with no signs of toxicity to the mice. The treated mice exhibited no weight loss compared to control mice, had normal liver histology and serum liver enzyme levels. Additionally, we treated mice with a siRNA derived from CAG trinucleotide repeats (TNRs) as found in Huntington’s disease (HD). We had shown earlier that CAG TNR siRNAs are much more potent in killing cancer cells in vitro than any other toxic si/miRNA we tested [29]. Repeated treatment with the highly potent siCAG caused significant reduction in tumor growth without any toxicity in the mice. Notably, the toxic siRNAs were delivered into mice systemically and were not designed to specifically target cancer cells. Therefore, the fact that normal tissues were unaffected suggests that in normal cells something prevented these toxic siRNAs from reaching the RISC or being functional.

A number of published studies demonstrate the efficacy and safety of employing RNAi to treat cancer, and their data are consistent with our model of 6mer Seed Tox. For instance, i.p. delivery of the tumor suppressive miR-193a-3p in liposomes substantially reduced the growth of HCT116, HT29, and MDA-MB-231 tumors when grown subcutaneously in xenografted mice [65]. Interestingly, both the 3p and the 5p arm of miR-193a contain highly toxic 6mer seeds (average viability of the 3p arm was 20.9% [seed: ACUGGC] and of the 5p arm 18.3% [seed: GGCUCU)], 6merdb.org). This miRNA is therefore a good candidate for reducing tumor growth through 6mer Seed Tox. Similar to our studies, these groups did not report any general toxicity in the treated mice.

We demonstrated that a large part of the antitumor activity of miR-34a-5p stems from its 6mer Seed [30]. Interestingly, in 2015, miR-34a-5p was the first miRNA to enter a phase I clinical trial. The trial included 154 patients with five different solid cancers. However, it was halted in 2016 after ~ 10% of treated patients exhibited adverse side effects due to elevated IL-6 levels. Yet, more general systemic toxicities, such as liver toxicity were not described [66]. It is unclear at present whether the observed side effects were caused by the delivery of unmodified naked miRNA, the liposomal particles, or activity of miR-34a-5p defined by a sequence outside its 6mer seed.

### Cancer selectivity of toxic siRNAs

Normal tissues may be less susceptible to toxic siRNAs than cancer cells. This notion is supported by a number of observations: 1) Treating OvCa bearing mice with either DISE-inducing siRNAs or CAG TNR-based siRNAs did not cause any toxicity in the treated mice [29, 64]; 2) Human normal ovarian fibroblasts immortalized with hTERT were more sensitive to 6mer Seed Tox than unmodified fibroblasts [24]; 3) Normal prostate epithelial cells are relatively insensitive to 6mer Seed Tox when compared to prostate cancer cell lines [31].

While there could be different explanations for the cancer selectivity of the toxic siRNAs, there are a number of not mutually exclusive mechanisms at play involving miRNA/RNAi pathway components that could influence the selectivity. Hence, we are proposing four different models:

1. *The miRNA protection model.* Drosha and Dicer KO cells are hypersensitive to various DISE-inducing stimuli [25, 30, 44, 58]. We interpreted this to be due to the loss of highly expressed miRNAs that contain nontoxic seeds. Without these nontoxic miRNAs, the KO cells may no longer be able to protect the RISC from loading of toxic sRNAs. This interpretation is supported by the following observations: 1) Titering a nontoxic siRNA into HeyA8 cells rendered them more resistant to the effects of toxic siRNAs [25]; 2) Drosha and Dicer KO cells take up toxic siRNAs much more readily into their RISC. As discussed above, this results in a dramatic shift in the average seed toxicity of RISC bound sRNAs from nontoxic to highly toxic [25, 29, 44, 58].

There is evidence that highly expressed miRNAs with nontoxic 6mer seeds protect normal cells from DISE induction. In most cells, more than 95% of all RISC-bound RNAs are miRNAs, with most miRNAs carrying nontoxic seeds [44]. In contrast, when most canonical miRNAs are absent as in Drosha and Dicer KO cells (Drosha and Dicer are required for miRNA biogenesis), the RISC is only partially occupied by miRNAs and is now more available to load toxic sRNA. This is why Dicer/Drosha KO cells could be hypersensitive to any DISE-inducing agent including siRNAs or miRNAs and to genotoxic drugs [25, 30]. This interpretation of our data is consistent with the observation that all cancer cells are characterized by a downregulation of miRNAs when compared to their matched normal tissue [53, 54]. Mechanistically this may occur through deregulation of Dicer or Exportin-5 expression [67], or through regulation of the activity of the Drosha/DGCR8 microprocessor complex [68].

2) *The differential Ago stability model.* Dicer or DGCR8 KO mice have demonstrated that in normal cells, such as embryonic stem cells or mouse embryonic fibroblasts, Ago proteins are degraded in the absence of miRNAs (discussed in [28, 69]). In contrast, both Dicer KO HCT116 and 293 T cells retain normal expression of Ago proteins [25, 30]. These transformed cells retain available RISC components to load toxic siRNAs. The fact that normal, untransformed cells degrade “naked” Ago proteins could explain their decreased sensitivity to DISE; When miRNA expression levels are low—as is the case in somatic stem cells—Ago protein levels are also reduced [70]. Thus, there is less opportunity to load non-canonical, toxic sRNAs into the RISC to trigger DISE.
3) *The genomic stability protection model*. Almost all genes involved in miRNA biogenesis, Drosha, DGCR8, Dicer, Exportin-5 (XPO5), and Ago2 have been found to be involved in the DNA damage response and/or DNA repair [22, 71–77]: DGCR8 plays a role in transcription-coupled nucleotide excision repair of UV-induced DNA lesions [77]; the catalytic activity of Ago2 is indispensable for its function in Rad51 recruitment and HR repair [75]; both Dicer and Drosha are required for DNA repair through the generation of Dicer and Drosha-dependent small RNAs (DDRNAs) [22], which facilitate recruitment of repair complexes to sites of DNA damage (also called DSB-induced small RNAs (diRNAs) [73]). In addition, Drosha also directly promotes nonhomologous end joining (NHEJ) by interacting with RAD50 [71]. Finally, Exportin-5 has been reported to indirectly promote genomic stability by exporting pre-miRNAs from the nucleus that facilitate DNA repair [76]. Because DISE-inducing siRNAs in part kill cancer cells by increasing ROS production and DNA damage [24], the sensitization of cells lacking any of the miRNA processing genes (e.g., the HCT116 mutant cells we tested) or RNAi mediating genes could also result from a reduced ability to repair DNA damage. It is known that cancer cells, due to their genomic instability and high ROS content, are vulnerable to certain genotoxic stresses [78].
4) *The survival gene dependence model.* It is conceivable that cancer cells, which in general proliferate at a higher rate than mostly quiescent normal cells, express survival genes at higher levels and hence are more dependent on their activities for cell survival than normal cells.

Regardless of the mechanism underlying the cancer selectivity of DISE, there is early evidence of a therapeutic window. By activating DISE in the correct context, one could kill cancer cells and spare normal cells. Further studies will seek to understand and extend this therapeutic window.

### Mechanisms of cancer therapy resistance

Despite recent therapeutic advances, recurrent ovarian cancer—particularly the most prevalent and most malignant form, high-grade serous ovarian cancer (HGSOC)—is incurable and has a poor prognosis, with a median survival of 40.7 months [79]. The main reason for ovarian cancer-related mortality, which is the highest of any gynecological cancer, is almost entirely due to the rapid emergence of resistance to platinum (Pt)-based chemotherapy [80].

miRNAs have been linked to cancer therapy resistance in countless reports in almost all cancers [81]. For ovarian cancer alone we found 118 publications reporting on at least 87 miRNAs that either confer acquired therapy resistance, re-sensitize ovarian cancer cells to treatment, or that correlate with or predict ovarian cancer treatment outcome (summarized in [44]). However, there is very little agreement or overlap in the identified miRNAs, raising the question of how this bewildering array of different miRNAs can be linked to therapy resistance in ovarian cancer.

We previously showed that treating cancer cells with chemotherapeutic drugs such as etoposide, carboplatin and doxorubicin caused upregulation of miRNAs with toxic 6mer seeds [30]. Indeed, many anticancer drugs including anthracyclines (e.g., doxorubicin, epirubicin), alkylating agents (e.g., cyclophosphamide), and platinum drugs (e.g., cisplatin, carboplatin, and oxaliplatin) kill cells in a manner similar to DISE-inducing siRNAs: they increase ROS levels and cause DNA damage [82, 83]. Thus, we explored the role of 6mer Seed Tox in therapy resistance in the context of ovarian cancer [44].

miRNAs in cancer are almost uniformly quantified using total RNA. However, our data suggest that, instead, it is more meaningful to analyze RISC-bound sRNAs given the fact that most mature miRNAs are not Ago associated [84] and that endogenous miRNAs vary widely in their level of RISC association [44, 85, 86]. We found that it is not the expression of any individual miRNA that may determine treatment outcome, instead it is the ratio of toxic-to-nontoxic RISC-bound sRNAs (mostly miRNAs) that determines cell fate. We found that in two pairs of platinum Pt sensitive and resistant ovarian cancer cell lines the Pt resistant cells contained more nontoxic miRNAs in the RISC than the Pt sensitive cells [44]. More importantly, in an analysis of primary ovarian cancer patient tumors (isolated at primary surgery), we found that Pt-resistant patients had a higher ratio of nontoxic-to-toxic miRNAs in their RISC than Pt sensitive patients. These data suggest that it may be possible to predict which patients will benefit the most from Pt-based chemotherapy based on their RISC content.

To provide a standardized tool to analyze 6mer Seed Tox, we recently developed SPOROS (Greek for seed), an automated bioinformatics pipeline for the analysis of small RNA Seq data [37]. SPOROS allows for the assessment of 6mer Seed toxicity of sRNAs in any cells or tissues by generating various graphical outputs (6mer seed graph, 6mer seed tox analysis, 6mer seed composition and others) [37].

While cancer cells can hardly become completely resistant to 6mer Seed Tox, they could however increase their tolerance through two possible mechanisms: 1) by altering the ratio of toxic-to-nontoxic sRNAs towards more nontoxic ones and 2) by acquiring mutations in the targeted 6mer seed matches. When we determined that G-rich seeds were the most toxic in the screen of the 4096 6mer seeds, we were surprised to note that C-rich seeds were much less toxic [30]. From a thermodynamic standpoint, GC-rich sequences should be more stable than AU-rich ones and it should not matter which one of these two nucleotides act in a seed versus a seed match. So why are G-rich seeds more toxic than C-rich ones? An answer to this question may lie in the different chemical stabilities of the two nucleotides. In contrast to guanines, cytosines are instable and can get lost by C-T transitions through deamination of cytosines which occurs through enzymatic deamination or spontaneous hydrolysis [87]. When only a single cytosine in a targeted seed match of a survival gene is converted to a thymine it can no longer be targeted by the same G-rich 6mer seed. Considering this, it is intriguing that across many cancers the most prevalent point mutation results in loss of cytosines. This observation is usually interpreted as a tendency for cancer cells to reduce the number of CpG islets that are subject to methylation events, methylation that often results in silencing of gene expression [88]. However, a gradual loss of cytosines could be causing an increased tolerance to DISE. Guanines may have been selected to be in the targeting sRNAs as it allows for broad targeting of hundreds of genes with multiple C-rich seed matches. Thus, the killing mechanism will remain mostly intact even when many cytosines are lost.

Single nucleotide polymorphisms (SNPs) represent another example of mutational changes that may affect disease outcome. SNPs are found in miRNAs and can occur in the miRNA seed [89, 90]. An example is miR-146a, a change in the seed sequence was proposed to influence disease outcomes in various cancers [91]. A detailed analysis studying the location of point mutations (both germline and acquired) in the seeds of all miRNAs is needed to determine the frequency at which SNPs change 6mer seed sequences.

In addition to these potential mechanisms affecting the activity of 6mer Seed Tox, another mechanism could involve the generation of toxic miRNAs, as this process must be tightly regulated at multiple levels. This could occur through RNA modifications or the changes in the expression or localization of RNA binding proteins. Dysregulation of these pathways in certain conditions such as advancing age could reduce the potency of the 6mer Seed Tox anti-cancer mechanism.

### Possible cancer age connection

It is clear that cancer incidence increases with age. Multiple mechanisms have been described to contribute to this phenomenon [92]. One established cause is the acquisition of somatic mutations. In fact, some of the mutations in the genome accumulate in a clock like fashion, increasing with age [93]. This discovery was made by analyzing more than 10,000 cancer genomes. 33 distinct mutational COSMIC signatures were identified. Only two of these signatures (1 and 5) showed a strong connection with age, irrespective of the type of cancer. It was the C > T transversion that showed the strongest association with age. While the analysis of cancer cells allowed for the identification of the clock like loss of cytosines in the genome, this also occurs in normal tissues. In fact, several reports have found that many normal tissues acquire a high rate of mutations with age, at times as high as in cancer cells [94–96]. Again, across all tissues, C > T mutations dominated the spectra.

As noted above, the loss of cytosines in the genome may hinder the anti-cancer function of 6mer Seed Tox. As cytosines in the 3′ UTR of survival genes are lost, this could decrease the probability that these genes are targeted by toxic G-rich sRNAs directing the RISC. Over time, the accumulation of C > T mutations could gradually relieve cells from 6mer seed Tox surveillance, resulting in the development of cancer. Thus, the accumulation, specifically of C > T mutations, provides a link between increased cancer incidence and old age through 6 mon Seed Tox.

### Potential relevance to neurodegenerative diseases

Assuming that normal cells are largely protected from 6mer Seed Tox by high concentrations of miRNAs with nontoxic seeds, this raises the question of what happens if a tissue loses this protection (Fig. 1B). We hypothesize that this would result in degeneration of the affected tissue. For example, the brain is an organ that loses miRNA expression with age [67]. Therefore, neurodegenerative disease may, in part, result from an increase in RISC-bound toxic sRNAs in the context of age-related miRNA downregulation. This change in the RISC paired with a concomitant increase in ROS levels [97] could sensitize even normal cells to DISE.

There is ample literature to support the notion that miRNAs are neuroprotective (reviewed in [69]). In fact, loss of miRNAs is associated with neurodegeneration. In *Drosophila* impairing miRNA processing dramatically enhanced neurodegeneration caused by the CAG repeat gene ATXN3 [98]. Multiple publications on mice with a brain specific knock-out of Dicer showed that reducing Dicer triggered neuronal loss and caused behavioral abnormalities, followed by the premature death of the animals (see e.g. [99, 100]). Moreover, mice with a tissue specific deletion of Dicer in spinal motor neurons exhibit hallmarks of spinal muscular atrophy (SMA) with signs of denervation [101]. Interestingly, mice with a brain specific knock-out of Ago2 (using the same promoter used to delete Dicer in the brain: CaMKII-Cre) showed no toxicity in neurons [102], suggesting that it is not the general activity of miRNAs and RNAi that is required for neuronal survival.

In fact, it has been shown that increasing miRNA levels can be neuroprotective. Enoxacin is an antibiotic drug that stabilizes Dicer/TRBP resulting in a global upregulation of miRNAs [103–105]. In a mouse model of Amyotrophic Lateral Sclerosis (ALS), treatment with Enoxacin resulted in increased miRNA expression and reduced disease scores [106]. Likewise, using Dicer KO mice it was directly shown that miRNAs protect adult dopamine (DA) neurons from neurotoxicity [107] and Enoxacin treatment promoted survival of cultured wild-type DA neurons exposed to ER stress. All these data are consistent with the notion that higher expression of miRNAs provides protection from neurodegeneration. We think that many neurodegenerative diseases could similarly be promoted through dysregulation of RNAi, including TNR expansion diseases such as HD (reviewed in [69]).

In summary, miRNA (or miRNA biogenesis enzyme) levels may drop in the aging brain resulting in a progressive loss of protective miRNAs. This in turn could lead to a relative increase in toxic sRNAs in the RISC (e.g., toxic CAG TNR based short CAG repeats [108] or G/C-rich tRNA fragments), resulting in the loss of neurons and progression of disease (Fig. 1B).

## Conclusions and outlook

DISE/6mer Seed Tox is a promising new approach to treat various cancers. While early preclinical data are encouraging, further testing is needed to determine whether loss of protective miRNAs underlies sensitivity to this type of cell death, and whether aged mice (or patients) exhibit signs of general toxicity, or whether other mechanisms underlie sensitivity. If miRNA downregulation underlies sensitivity, 6mer Seed Tox could be relevant in additional disease contexts. For example, the tissues of the spleen and adipose tissue have also been shown to lose expression of Dicer and/or Exportin-5 with age, and thus may also have globally downregulated miRNA levels [67]. Therefore, diseases that involve immune cells (such as autoimmune diseases), or adipocytes (such as type II diabetes) could also result, in part, from increased sensitivity to 6mer Seed Tox. Previously, we published evidence to suggest that many viral (v-)miRNAs have the capacity to kill cells through 6mer Seed Tox [45]. We transfected 215 v-miRNAs encoded by 17 human pathogenic viruses into cells and found that many v-miRNAs including HIV-1 miRNAs, could be toxic through 6mer Seed Tox. Interestingly, it has been reported that with age the effects of HIV-1 infection can become more severe [109]. In general, any tissue with a reduced ability to form protective miRNAs, either due to the downregulation of components of the miRNA biogenesis machinery or because of increased genomic stress, could be susceptible. This decreased ability to generate miRNAs may be related to age, infection, inflammation, or other triggers. While these scenarios are speculative, they can now be tested experimentally given the framework provided in this review.

## Data Availability

Not applicable.

## Abbreviations

DISE: Death induced by survival gene elimination
UTR: Untranslated region
RISC: RNA induced silencing complex
6mer Seed Tox: 6mer seed toxicity
RNAi: RNA interference
miRNA: microRNA
dsRNA: Double stranded RNA
Ago: Argonaute
AR: Androgen receptor
AN-DISE: Androgen network death induced by survival gene elimination
CLIP: Crosslinking immunoprecipitation
siRNA: Small interfering RNA
shRNA: Short hairpin RNA
KO: Knock-out
sRNA: Short RNA
TNR: Trinucleotide repeat
HD: Huntington’s disease
CD95L: CD95 ligand
HGSOC: High-grade serous ovarian cancer
Pt: Platinum
DDRNAs: Dicer and Drosha-dependent small RNAs
diRNAs: DSB-induced small RNAs
